# Anaemia in pregnant adolescent girls with malaria and practicing pica

**DOI:** 10.11604/pamj.2016.24.96.9282

**Published:** 2016-05-27

**Authors:** Freda Dzifa Intiful, Edwin Kwame Wiredu, George Awuku Asare, Matilda Asante, David Nana Adjei

**Affiliations:** 1 School of Biomedical and Allied Health Sciences, University of Ghana, Ghana

**Keywords:** Pregnant, adolescent, anaemia, pica, malaria, Ghana

## Abstract

**Introduction:**

Pregnancy during the adolescent period is challenging mainly because of the nutritional demands of both the adolescent and pregnancy period. The risk for anaemia increases especially in developing countries such as Ghana where malaria is endemic and the practice of pica is common. In this study, we sought to determine the prevalence of anaemia, pica practice and malaria infection among pregnant adolescent girls and assess the extent to which these factors are associated.

**Methods:**

Two hundred and sixty five (265) pregnant adolescent girls were recruited from three hospitals in Accra. Haemoglobin levels, malaria infection and the practice of pica were assessed. Pearson's Chi squared tests were used to determine associations and logistic regression analysis was used to determine the odds of being anaemic. Significance was set at p≤0.05.

**Results:**

Anaemia prevalence was 76% with severity ranging from mild (47.8%) to severe (0.8%). About 27.5% were moderately anaemic. Pica was practiced in only 9.1% of the girls. Malaria infection was prevalent in 17.7% of the girls. The logistic regression analysis indicated that pregnant girls with malaria infection were 3.56 times more likely to be anaemic when compared to those without malaria. Also, those who practiced pica were 1.23 times more likely to be anaemic when compared to those who did not practice pica.

**Conclusion:**

Anaemia is very prevalent in pregnant adolescent girls and is a public health problem. Drastic measures should be taken to reduce the high prevalence.

## Introduction

The adolescent period is characterized by intense growth physically, psychosocially and cognitively. The intensity of this growth period necessitates the increase in nutritional needs. During this period there is a high demand for iron, especially in the adolescent girl because of the onset of mensuration. Iron deficiency is a major cause of anaemia. The risk for anaemia increases when the adolescent girl becomes pregnant. There are several factors that interact and contribute negatively to the outcome of pregnancy in the adolescent girl. The presence of anaemia, practice of pica and malaria has all been implicated. The presence of these conditions in pregnant adolescent girls can pose greater challenge because of the obvious burden of the pregnancy on the rapidly growing mother which additionally has to cater for another developing foetus. Anaemia affects about 38% of pregnant women globally. The prevalence is about 44.6% in Africa and 56% in Ghana [1]. The World Health Organization(WHO) considers this prevalence to be unacceptable and of severe public health significance [1]. Anaemia in pregnancy is of great concern as it is associated with complications such as low birth weight, preterm delivery, spontaneous abortions and ultimately death [2, 3]. There is also an established relationship between pica and anaemia [4, 5]. Pica is a condition involving the consumption of non-food substances. This practice has been found to be common among pregnant women even though it can also be seen among all categories of people including children. Several studies have linked the practice of pica to negative health outcomes such as micronutrient deficiencies particularly iron, zinc and calcium [6].

Malaria is an endemic infectious disease and a common public health problem in Ghana, affecting pregnant women. It is globally ranked as the second most common cause of deaths related to infectious disease after tuberculosis [7]. According to Takem and D'Alessandro [8] the presence of malaria infection in pregnant women is higher than in non- pregnant women probably resulting from the several changes that occurs during the period of pregnancy; including hormonal and immunologic factors making pregnant women more susceptible. In a recent review, malaria infection among pregnant women across West and Central Africa was reported to be about 35.1% (95%CI: 28.2–41.9) [9]. There are severe consequences of malaria infection in pregnancy. These include anaemia, miscarriage, maternal mortality, low birth weight and still birth [7]. The WHO estimates that malaria infection accounts for more than 10,000 and 100, 000 maternal and neonatal deaths respectively [10]. In Ghana, adolescent pregnancy accounts for about 14% of all pregnancies. Maternal and infant mortality are still very high. Maternal mortality currently stands at 319 per 100, 000 live births and infant mortality 43 per 1,000 live births [11]. Therefore there is the need to ensure that all categories of mothers receive optimum care to help reduce maternal and child mortality as the nation strives to meet the target set by the Sustainable Development Goals. The aim of this study was to determine the prevalence of anaemia, pica practice and malaria infection among pregnant adolescent girls and assess the extent to which these factors are related.

## Methods

The study was a cross sectional study carried out among pregnant adolescent girls between the ages of 15-19 years. The girls were attending ante-natal clinics for the first time at three hospital facilities in the Greater Accra Region of Ghana namely the Mamprobi Polyclinic, James Town Maternity Home (Ussher fort Polyclinic) and the La Polyclinics. A total of 265 pregnant adolescent girls were recruited for the study. All pregnant girls who reported for the first time for ante-natal clinic were approached and invited to be part of the study. Those who consented and were apparently healthy were recruited. Pregnant girls who had cases of haemoglobinopathies were excluded from the study. Ethics approval for the research was obtained from the Ethics and Protocol Review Committee of the School of Biomedical and Allied Health Sciences of the University of Ghana, College of Health Sciences. Permission was also sought from the Greater Accra Health Directorate and the health facilities where the study was undertaken.

### Data collection

Semi-structured questionnaires were used to obtain information regarding patient's background including ages, level of education, marital status and practice of pica obtained. Five (5) ml of blood was drawn into an EDTA tube. Full blood count was performed to determine haemoglobin levels. Cut-off values for the determination of anaemia in pregnancy were set based on World Health Organization (WHO) criteria [12]. The following categories for anaemia were used: Hb<7g/dl –Severe, Hb 7.0-9.9- Moderate, Hb 10-10.9- Mild, and Hb > 11- Normal. A rapid test kit method, Coretests^®^ One Step Malaria Pf/Pv Ag Test kit(Core Technology: Beijing, China)was used to determine the presence of malaria infection according to manufacturer's instructions. Data were entered and analysed using SPSS version 17 and summarized using means and percentages. Pearson's Chi squared tests were used to determine associations and logistic regression analysis was used to determine the odds of being anaemic. The level of significance was set at p = 0.05 with a confidence interval of 95%.

## Results

The background characteristics of the girls are summarized in Table 1. The mean age of the girls was 17.88; (SD=1.08) years. The 18 year olds formed the majority (37.7%) of the girls; followed by the 19 year olds (33.2%) with the 15 year olds (4.2%) being the least represented. More of the girls (63%) were in their second trimester with only 8.7% in their third trimester. The highest level of education attained by the girls was senior high school level (2.6%). Majority of them had junior high school (36.2%) and primary (33.6%) education as the highest educational level attained. About 27.5% of them had no form of education. Almost half of the girls (47.5%) were unemployment. The main form of employment was petty trading (33.6%). About 89% of the girls were single with only 3.8% indicating they were married. The rest (6.8%) were co-habiting with their partners. Table 2 describes the prevalence of anaemia, malaria infection and pica practice among the pregnant girls. The mean haemoglobin concentration was 10.32 (SD=1.32) g/dl. Anaemia based on haemoglobin status was very high among the girls. About 76% of the girls were anaemic. Anaemia severity ranged from mild (47.8%) to severe (0.8%). Moderate anaemia was 27.5%. Anaemia was more prevalent among those in the second trimester (79.4%) compared to those in the first (72%) and third (69.5%) trimesters. The evaluation of the practice of pica indicated that about 9% of the girls practiced some form of pica. Pica was more prevalent in the third trimester (30.4%) compared to the other trimesters. Malaria parasites were identified in 17.7% of the girls. The association between anaemia, malaria infection and pica practices are presented in Table 3, Table 4. Pearson's Chi-square tests showed statistically significant association between anaemia and malaria infection (χ^2^13.91, p = 0.003, df =3) and anaemia and pica practices (χ^2^13.26, p = 0.004, df=3). Majority (51.3%) of those who had malaria were moderately anaemic (Table 3). About 46% of those practicing pica were mild to moderately anaemic (Table 4). The percentage of girls who practiced pica and were anaemic and those with malaria infection and anaemic are presented in Table 5. Most of the girls (83.3%) practicing pica were anaemic whiles 46.2% of those with malaria infection were also anaemic. A logistic regression analysis was performed to predict the likelihood of anaemia (Table 6). The results indicated that the girls infected with malaria infection were 3.56 times more likely to be anaemic as compared to those who were not infected. Also, those who practiced pica were 1.23 times likely to be anaemic compared to those who did not practice pica. When the model was adjusted for age, trimester and education level, the odds of being anaemic slightly increased for both girls infected with malaria infection and those practicing pica.

**Table 1:** Background information of pregnant adolescent girls

Variable	1st Trimester N (%)	2^nd^ Trimester N (%)	3^rd^ trimester N (%)	Total N (%)
***Age (years)*** Mean ±SD (17.88±1.08)				
15	6 (2.3)	4 (1.5)	1 (0.4)	11 (4.2)
16	1 (0.4)	19 (7.2)	0 (0.0)	20 (7.5)
17	15 (5.7)	24 (9.1)	7 (2.6)	46 (17.4)
18	27 (10.2)	66 (24.9)	7 (2.6)	100 (37.7)
19	26 (9.8)	54 (20.4)	8 (3.0)	88 (33.2)
**Total**	**75 (28.3)**	**167 (63.0)**	**23 (8.7)**	**265 (100)**
***Educational level***				
No education	19 (7.2)	48 (18.1)	6 (2.3)	73 (27.5)
Primary	26 (9.8)	56 (21.1)	7 (2.6)	89 (33.6)
Junior secondary	29 (10.9)	57 (21.5)	10 (3.8)	96 (36.2)
Senior secondary	1 (0.4)	6 (2.3)	0 (0.0)	7 (2.6)
**Total**	**75 (28.3)**	**167 (63.0)**	**23 (8.7)**	**265 (100)**
***Occupation***				
Unemployed	39 (14.7)	75 (28.3)	12 (4.5)	126 (47.5)
Artisan	3 (1.1)	11 (4.2)	3 (1.1)	17 (6.4)
Trader	25 (9.5)	58 (21.9)	6 (2.3)	89 (33.6)
Other	8 (3.0)	23 (8.7)	2 (0.8)	33 (12.5)
**Total**	**75 (28.3)**	**167 (63.0)**	**23 (8.7)**	**265 (100)**
***Marital status***				
Married	6 (2.3)	2 (0.8)	2 (0.8)	10 (3.8)
Single	65 (24.5)	155 (58.5)	17 (6.4)	237 (89.4)
Co habitation	4 (1.5)	10 (3.8)	4 (1.5)	18 (6.8)
**Total**	**75 (28.3)**	**167 (63)**	**23 (8.7)**	**265 (100)**

**Table 2:** Anaemia prevalence, pica practice and malaria infection among pregnant adolescent girls

Variables	1^st^ trimester (N=75) n (%)	2^nd^ trimester (N=167) n (%)	3^rd^ trimester (N=23) n (%)	Total (N= 265) n(%)
***Haemoglobin status*** **(g/dl)** **(Mean ±SD)**				
Normal (11.97±0.80)	12 (16.0)	35 (20.96)	7 (30.4)	63 (23.8)
Mild anaemia (10.40±0.23)	37 (49.3)	80 (47.9)	10 (43.5)	127 (47.8)
Moderate anaemia (8.88±0.78)	17 (22.7)	50 (30.30)	6 (26.1)	73 (27.5)
Severe anaemia (5.40±0.28)	0 (0.0)	2 (1.2)	0 (0.0)	2 (0.8)
**Total** (10.32±1.32)	**75 (100.0)**	**167 (100.0)**	**23 (100.0)**	**265 (100.0)**
***Pica practice***				
Yes	4 (5.3)	13 (7.8)	7 (30.4)	24 (9.1)
No	71 (94.7)	154 (92.2)	16 (69.60)	241 (90.9)
**Total**	**75 (100.0)**	**167 (100.0)**	**23 (100.0)**	**265 (100)**
***Malaria infection***	**(N=66)**	**(N=136)**	**(N=18)**	**(N=220)**
Yes	6 (9.1)	29 (21.3)	4 (22.2)	39 (17.7)
No	60 (90.9)	107 (78.7)	14 (77.8)	181 (82.3)
**Total**	**66 (30.0)**	**136 (61.8)**	**18 (8.2)**	**220 (100)**

**Table 3:** Association between malaria infection and anaemia (N=220)

Malaria status	Normal haemoglobin n (%)	Mild anaemia n (%)	Moderate anaemia n (%)	Severe Anaemia n (%)	Total N (%)
Infected	6 (15.4)	12 (30.8)	20 (51.3)	1 (2.6)	39 (100)
Not Infected	48 (26.5)	89 (49.2)	43 (23.8)	1 (0.6)	181 (100)

χ^2^(3, N=220)=13.91, *p*=0.003

**Table 4:** Association between anaemia and pica practice

Pica practice	Normal haemoglobin n (%)	Mild anaemia n (%)	Moderate anaemia n (%)	Severe anaemia n (%)	Total N (%)
**Yes**	13 (54.2)	7 (29.2)	4 (16.7)	0 (0.0)	24 (100)
**No**	50 (20.7)	120 (49.8)	69 (28.6)	2 (0.8)	241 (100)

χ^2^(3, N=265)=13.26, *p*=0.004

**Table 5:** Percentage of pregnant girls practicing pica and with malaria infection

Haemoglobin status	Pica (N=24) n(%)	Malaria infection (N=39) n(%)
**Normal**	4 (16.7)	21 (53.8)
**Anaemic**	20 (83.3)	18 (46.2)
**Total**	24 (100.0)	39 (100.0)

**Table 6:** The odds of being anaemic among pregnant adolescent girls

Factor	Unadjusted			Adjusted		
	Odds ratio	95% CI	p-value	Odds ratio	95% CI	p-value
**Pica Practice**						
Yes	1.23	0.39-3.92	0.727	1.30	0.39-4.4	0.618
No	1.00	(Reference)		1.00	(Reference)	
**Malaria infection**						
Infected	3.56	1.73-7.34	0.001*	3.62	1.72-7.56	.001*
Not infected	1.00	(Reference)		1.00	(Reference)	

* Significance p<0.05

## Discussion

Anaemia in pregnancy is an obstetric risk which needs to be treated to prevent complications. The present study reports a disturbingly high (76.2%) prevalence of anaemia among pregnant girls. This is higher than the national prevalence of 56% in pregnant women in Ghana and other reported studies in Kenya (61%), India (63%) and Nigeria (40.4%) [1, 13–15]. The presence of anaemia among pregnant women is not a new phenomenon but the severity of the prevalence among this population of adolescent girls is alarming. This high prevalence can be explained not only by the physiologic demands during pregnancy and the adolescent period but other factors including low dietary intakes of nutrients such as iron can result in deficiencies and worm infestations. As a matter of policy, pregnant women attending antenatal care in Ghana are mandatorily supplemented with haematinics to reduce the incidence of anaemia. The girls in this present study were recruited on the first day of attending antenatal clinic and therefore it is more likely that a large number of them would not be on any supplements that would help curb the anaemia situation. Pica is one of the factors that has been identified as a risk to developing anaemia in pregnancy [16]. Contrary to other studies this present study found a low (9%) practice of pica among the girls. This finding was intriguing when compared to other studies. The prevalence was relatively lower than 47% reported elsewhere in Ghana, 44%, 46% and 74% reported in Mexico, US and Kenya respectively [17–20]. Even though participants in this study may not have received any education from health professionals at the time of participating in the study, it is possible they might have heard of the dangers of consuming these substances from some other sources such as the media and this could have contributed to their low practice of pica. Majority of the girls practicing pica (83.3%) were found to be anaemic. When a logistic regression was performed, they were 1.23 times more likely to be anaemic. The relationship between pica and anaemia has been documented in other studies [5, 21]. Therefore, in spite of the low prevalence reported in this study, it is still prudent to educate pregnant women and adolescents against practicing pica because of its reported link to anaemia in pregnancy and incidence of lead poisoning [22].

Malaria infection was present in about 17.7% of the girls. In similar studies Uddenfeldt et al [23] and Glover-Amengor et al [24] reported 41% and 35% among their study participants. Ofori etal [25] also reported 19.7% malaria infection among pregnant women at the time of enrolment into the study whiles Tay et al [26] reported 12.6%. The disparities in the prevalence could be explained with an assertion by Ofori et al [25]. According to Ofori et al, there is a considerable level of variation in the incidence of malaria infection across the year. The highest incidence usually occurring after the rains therefore the incidence could be affected by the period the data were obtained. It is also important to note that the girls in this study may not be on any prophylactic treatment that is usually given to pregnant girls when they go for antenatal clinic because they were recruited in the study when they had just reported at the antenatal clinics for the first time. It is therefore possible that the continuous education on malaria in the media could have informed them in protecting themselves against the infection. Consistent with other findings, this study reported significant association between anaemia and pica practices and also anaemia and malaria infection. When a logistic regression was performed, those infected were more than three times likely to be anaemic. The likelihood slightly increased when the model was further adjusted by age, trimester and education level. This is consistent with other findings where malaria infection has been linked to the presence of anaemia in pregnant women [24, 27]. Due to the adverse effects of malaria infection in pregnancy, recommendations have been made to use insecticide treated mosquito nets and prophylactic drugs such as sulfadoxine pyrimethamine. The use of these measures have brought reductions in anaemia and low birth weight delivery in pregnant women [28, 29]. There were some limitations to this study. The design of the study was cross sectional and therefore causality could not be assumed. Other factors that may also contribute to the prevalence of anaemia such as iron deficiency, folate deficiency and worm infestation were not considered in this paper.

## Conclusion

In conclusion, this study has demonstrated that anaemia prevalence in adolescent pregnant girls is high. Pregnant girls who were anaemic were more likely to be practicing pica and infected with malaria. This report should further strengthen the need to investigate pica practices in pregnant adolescent girls during antenatal clinics and also step up measures to prevent malaria infections.

### What is known about this topic

- Prevalence of anaemia in all pregnant women in Ghana;
- Malaria infection among women who are pregnant in Ghana.

### What this study adds

- Prevalence of anaemia in pregnant adolescent girls in Ghana;
- The prevalence of the practice of pica among pregnant adolescent girls;
- The contribution of malaria and pica in anaemia among pregnant adolescent girls.
